# PFKFB3 Control of Cancer Growth by Responding to Circadian Clock Outputs

**DOI:** 10.1038/srep24324

**Published:** 2016-04-15

**Authors:** Lili Chen, Jiajia Zhao, Qingming Tang, Honggui Li, Chenguang Zhang, Ran Yu, Yan Zhao, Yuqing Huo, Chaodong Wu

**Affiliations:** 1 Department of Stomatology, Union Hospital, Tongji Medical College, Huazhong University of Science and Technology, Wuhan, Hubei 430022, China; 2 Department of Nutrition and Food Science, Texas A&M University, College Station, TX 77843, USA; 3 Vascular Biology Center, Department of Cellular Biology and Anatomy, Medical College of Georgia, Georgia Regents University, Augusta, GA 30912, USA; 4 Drug Discovery Center, Key Laboratory of Chemical Genomics, Peking University Shenzhen Graduate School, Shenzhen 518055, China

## Abstract

Circadian clock dysregulation promotes cancer growth. Here we show that PFKFB3, the gene that encodes for inducible 6-phosphofructo-2-kinase as an essential supporting enzyme of cancer cell survival through stimulating glycolysis, mediates circadian control of carcinogenesis. In patients with tongue cancers, PFKFB3 expression in both cancers and its surrounding tissues was increased significantly compared with that in the control, and was accompanied with dys-regulated expression of core circadian genes. In the *in vitro* systems, SCC9 tongue cancer cells displayed rhythmic expression of PFKFB3 and CLOCK that was distinct from control KC cells. Furthermore, PFKFB3 expression in SCC9 cells was stimulated by CLOCK through binding and enhancing the transcription activity of PFKFB3 promoter. Inhibition of PFKFB3 at zeitgeber time 7 (ZT7), but not at ZT19 caused significant decreases in lactate production and in cell proliferation. Consistently, PFKFB3 inhibition in mice at circadian time (CT) 7, but not CT19 significantly reduced the growth of implanted neoplasms. Taken together, these findings demonstrate PFKFB3 as a mediator of circadian control of cancer growth, thereby highlighting the importance of time-based PFKFB3 inhibition in cancer treatment.

Circadian clocks drive daily rhythms and coordinate many bio-physiological processes of living organisms to match environmental light-dark cycles^1^,^2^. This role of circadian clocks is achieved through clock control of rhythmic expression of genes in a wide range of tissues (cells)^3^,^4^. The circadian clock-controlled genes (CCG), for example, include those that are related to cell cycle such as c-Myc, p53, and cyclin D1, DNA modifications such as XPA, and metabolism such as glucose-6-phosphatase, peroxisome proliferator-activated receptor, and nicotinamide phosphoribosyltransferase^5^,^6^. As supported by many clinical investigations, the dysregulation of core clock genes is common in cancer tissues^7^,^8^,^9^,^10^. This appears to contribute to cancer development and promote cancer progression, likely by directly and indirectly dys-regulating CCG^11^,^12^,^13^,^14^. As additional evidence, approximate 4–6% CCG that are related to cell cycle, apoptosis, and metabolism displayed dysregulation of rhythmic expression^15^,^16^,^17^,^18^.

The link between circadian clock dysregulation and cancer development and progression implies the feasibility of timed anti-cancer therapies in order to generate maximal therapeutic effects and minimal unwanted-side effects^19^,^20^,^21^,^22^. This feasibility has been validated, at least, by the findings that circadian rhythm-dependent intervention, e.g., timed cyclin-dependent kinase suppression or meal timing^23^,^24^, differentially influenced the efficiency of cancer inhibition. Additionally, circadian variation of cell-cycle proteins in human oral mucosal biopsies led to a study to compare the toxicity associated with timed radiotherapy of patients with head-and-neck cancer^25^,^26^, which demonstrated that morning radiotherapy was associated with apparent reduction of mucositis and less weight loss compared with afternoon radiotherapy. Similarly, anti-metabolism-based evening chemotherapy for children with acute lymphoma resulted in a significant increase in 5-year survival rate compared with morning chemotherapy^27^,^28^. Together, these studies clearly demonstrate circadian clocks as important targets for effective cancer intervention. However, it remains largely unknown whether and how metabolism is dysregulated in the context of cancer pathophysiology; although metabolism oscillates in response to circadian clock outputs.

At the cellular level, the growth of most cancer cells displayed increased rates of glycolysis, where a regulatory step is controlled by PFKFB3, the gene that encodes for inducible 6-phosphofructo-2-kinase (iPFK2). The latter generates fructose-2,6-bisphosphate to stimulate glycolysis mainly through activating glycolytic enzyme 6-phosphofructo-1-kinase^29^,^30^. As a pro-cancer gene, PFKFB3 has been shown to critically control cancer cell survivals. Indeed, the association between PFKFB3 and cancer growth was first established in a human study by Atsumi *et al.*^31^; although the Warburg effect was discovered in 1920 s^32^. In cancer cells, an increase in glycolysis, resulted from the elevated PFKFB3 expression and/or iPFK2 activity, can at least 1) provide ATP to support physiological needs of cancer cells; 2) decrease pH value of microenvironment which contributes to increased apoptosis of normal cells; and 3) supply biosynthetic precursors such as fructose-6-phosphate and glyceraldehyde-3-phosphate for *de novo* nucleic acid synthesis. Because of this, inhibiting PFKFB3 has been considered an anti-cancer therapy^33^. To date, there is no research addressing whether PFKFB3 expression is rhythmic and whether cancerous cells and normal cells display different PFKFB3 rhythmicity. The latter, if exists, could provide a basis for novel anti-cancer therapy through timed PFKFB3 inhibition. The present study investigated the link between circadian clock dysregulation and PFKFB3 expression in human tongue cancer and tongue cancerous cells, and demonstrated a critical role for PFKFB3 in controlling tongue cancer by responding to circadian clock outputs.

## Results

### Increased PFKFB3 expression is accompanied with dysregulation of core clock gene expression in human tongue cancer tissues

**Figure 1:**
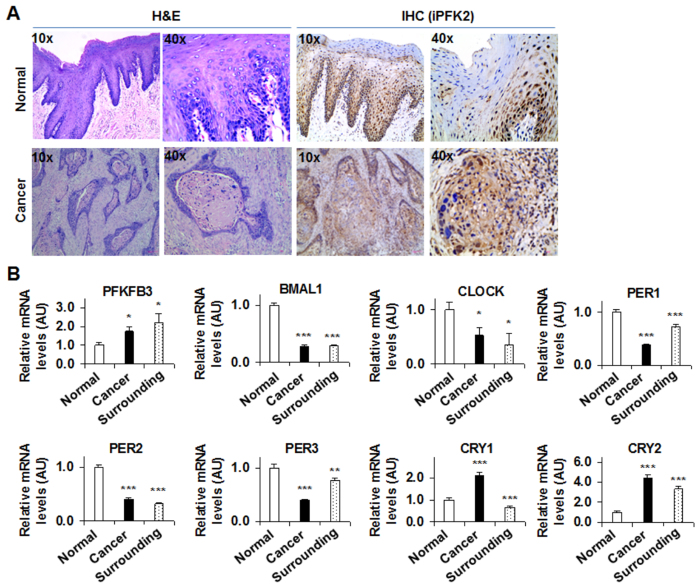
Human tongue cancer is associated with increased PFKFB3 expression and the dysregulation of core clock genes. Samples of human tongue cancers were obtained as described in Materials and Methods, which included tongue squamous cancer tissue (n = 27), matched tumor adjacent (surrounding tissues) (n = 27), and benign tissues (n = 28). (**A**) Tissue sections were used for H&E staining (left four panels) and for immunohistochemical staining to examine the amount of iPFK2, the enzyme encoded by PFKFB3 (right four panels). Top panels, normal tissues; bottom panels, cancer tissues. (**B**) The mRNA levels of PFKFB3, as well as core clock genes were quantified using real-time PCR and plotted as relative expression. Data are means ± SE. ^*^P < 0.05 and ^***^P < 0.001 vs. Normal.

PFKFB3 is a pro-cancer gene. The present study examined PFKFB3 expression in human tongue cancer tissues at both protein and mRNA levels. Subjects with or without tongue cancers displayed nearly identified clinical aspects including age and general lab biochemical parameters (Table 1). In surgically removed tissue samples, tongue cancer was validated using H&E staining (Fig. 1A). As indicated by the results from immunohistochemical staining, tongue cancer samples showed a significant increase in iPFK2 amount compared with normal tongue tissues (Fig. 1A). In addition, the dark brown sediments, indicating iPFK2, were mainly assembled in the basal layer of squamous cancer tissues, where original pleomorphic cancer cells were found frequently. Inside cancer cells, the dark brown sediments were mainly observed in the cytoplasm. Next, the mRNA levels of PFKFB3, along with several core clock genes were analyzed. Compared with those in normal epithelium, the mRNA levels of PFKFB3 were increased in both cancer tissues and its surrounding tissues, which were accompanied with significant decreases in the mRNA levels of BMAL1 and CLOCK in the same tissues (Fig. 1B). In addition, the expression of key clock genes whose products generate feedback inhibitory effects on BMAL1/CLOCK transcription activity in cancer tissues and its surrounding tissues was distinct from that in normal tissues. These genes include Period1 (PER1), PER2, and PER3, as well as Cryptochrome 1 (CRY1) and CRY2 (Fig. 1B). These results suggest that PFKFB3 expression is increased and associated with the dysregulation of core clock gene expression in human tongue cancer.

### Tongue cancerous cells display distinct PFKFB3 expression rhythmicity

**Figure 2:**
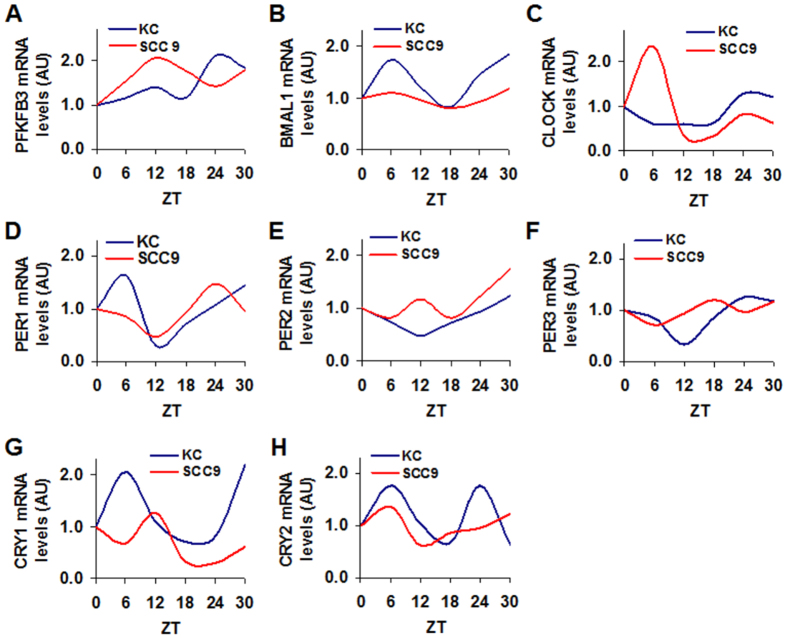
Cancer cells display distinct rhythmic expression of PFKFB3 and core clock genes. Human tongue cancer SCC9 cells and control KC cells were used to measure rhythmic expression of PFKFB3, as well as core clock genes. After synchronization, cells were incubated for an additional time points (zeitgeber time) as indicated and harvested for preparation of total RNA. The mRNA levels of PFKFB3, as well as BMAL1, CLOCK, PER1, PER2, PER3, CRY1, and CRY2 were quantified using real-time PCR and plotted as relative expression. Data are means. n = 3. (**A**) PFKFB3 oscillation. (**B**) BMAL1 oscillation. (**C**) CLOCK oscillation. (**D**) PER1 oscillation. (**E**) PER2 oscillation. (**F**) PER3 oscillation. (**G**) CRY1 oscillation. (**H**) CRY2 oscillation.

Considering that the clinical samples were collected at various daytime points, the present study also compared the rhythmicity of PFKFB3, as well as core clock genes in synchronized cancerous cells and control cells. In relative to control KC cells, human tongue cancer SCC9 cells displayed a different pattern of rhythmic PFKFB3 expression (Fig. 2A), which was characterized by a mid-phase peak in PFKFB3 expression. With regard to BMAL1 and CLOCK rhythmicity, SCC9 cells showed a robust peak in CLOCK expression but not in BMAL1 expression whereas KC cells showed a peak in BMAL1 expression but not in CLOCK expression (Fig. 2B,C). In addition, KC cells displayed typically rhythmic expression in PER1, PER2, and PER3, as well as CRY1 and CRY2 (Fig. 2D–H). In SCC9 cells, rhythmic expression of PER1, PER2, PER3, and CRY1 appeared to be lagged behind in phase of peaked expression. SCC9 cells also lacked a late-phase peak in CYR2 expression compared with KC cells (Fig. 2H). These results suggest that tongue cancerous cells are distinct from control cells in rhythmic expression of PFKFB3, as well as core clock genes.

**Figure 3:**
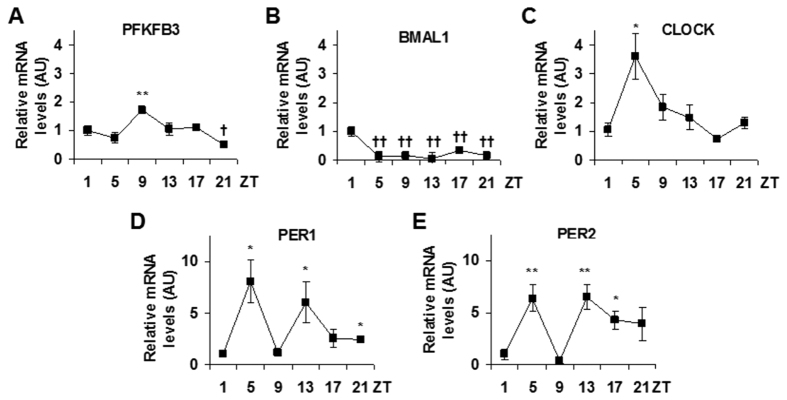
Cancer cells reveal rhythmic expression of PFKFB3 and core clock genes. Human tongue cancer SCC9 cells were used to measure 24 hr oscillation of the expression of PFKFB3, as well as core clock genes. After synchronization, cells were incubated for an additional 1, 5, 9, 13, 17, and/or 21 hr (zeitgeber time, ZT1, ZT5, ZT9, ZT13, ZT17, and/or ZT21) and harvested for preparation of total RNA. The mRNA levels of PFKFB3, as well as core clock genes were quantified using real-time PCR and plotted as relative expression. Data are means ± SE. (**A**) PFKFB3 oscillation. (**B**) BMAL1 oscillation. (**C**) CLOCK oscillation. (**D**) PER1 oscillation. (**E**) PER2 oscillation. Data are means ± SE. n = 6. *P < 0.05 and **P < 0.01 vs. ZT1 for increased expression of the same gene; ^†^*P* < 0.05 and ^††^*P* < 0.01 vs. ZT1 for decreased expression of the same gene.

Next, the present study focused on SCC9 cells to further examine the relationship between PFKFB3 and key clock genes. In synchronized cells, the mRNA levels of PFKFB3 and core clock genes were measured at 1, 5, 9, 13, 17, and 21 hr after synchronization (Zeitgeber time, ZT1, ZT5, ZT9, ZT13, ZT17, and ZT21). At the mRNA level, PFKFB3 expression displayed obvious 24-hr oscillation (Fig. 3A), which was similar to that of CLOCK (Fig. 3C). In particular, both PFKFB3 and CLOCK were increased in early phase during the 24 hr time period and reached peak levels between ZT5 and ZT9, and decreased in a similar pattern in late phase and reached the lowest levels between ZT17 and ZT21. In relative to CLOCK expression, PFKFB3 expression was peaked at a time point lagged about 4 hr bind CLOCK peak expression. However, BMAL1 expression, as well as PER1 and PER2 expression displayed apparent differences in rhythmicity compared with PFKFB3 and/or CLOCK (Fig. 3B,D,E). Taken together, these results indicate a close relationship between PFKFB3 and CLOCK.

### CLOCK increases transcription activity of PFKFB3 promoter in cancer cells

**Figure 4:**
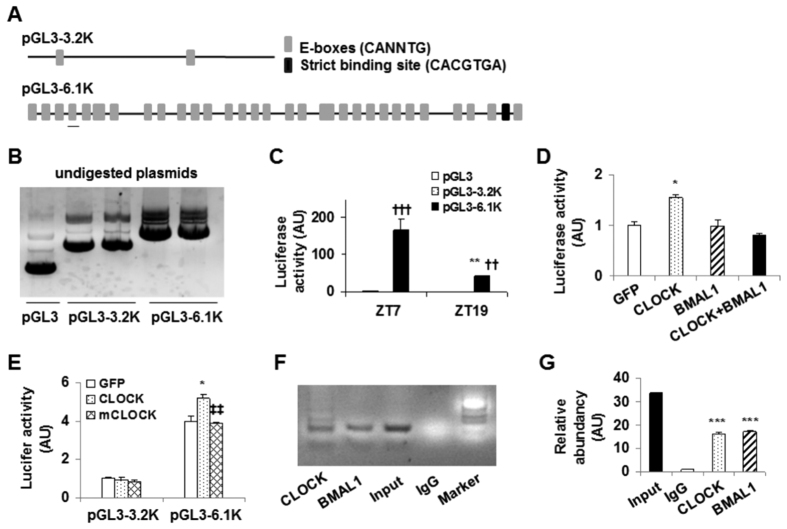
CLOCK enhances PFKFB3 expression. The promoter -reporter assay and ChIP assay were performed in SCC9 cells. (**A**) Schemes of a 6.1 kb PFKFB3 promoter and a 3.2 kb irrelevant DNA fragment. The strict binding site for BMAL1/CLOCK is about 431 bp upstream of the starter codon of PFKFB3 DNA. A genuine E-box validated by the ChIP assay (**F**,**G**) was underlined. (**B**) Verification of reporter constructs. Un-digested plasmids were used for DNA gel electrophoresis. pGL3-6.1K, luciferase expression driven by a 6.1 kb fragment of PFKFB3 promoter; pGL3-3.2K, luciferase expression driven by a 3.2 kb irrelevant DNA fragment, and pGL3, a basic reporter construct. (**C**) Effects of endogenous clocks on PFKFB3 promoter activity. SCC9 cells were synchronized and transfected with pGL3-6.1K, pGL3-3.2K, or pGL3 at 7 hr and/or 19 hr post synchronization (zeitgeber time, ZT7 and/or ZT19). (**D**) CLOCK stimulation of PFKFB3 promoter transcription activity. SCC9 cells were co-transfected with pGL3-6.1K and BMAL1-, CLOCK-, or green fluorescent protein (GFP)-expressing plasmid. Some cells were co-transfected with pGL3-6.1K, BMAL1-expressing plasmid, and CLOCK-expressing plasmid. (**E**) Effects of mutant CLOCK on PFKFB3 promoter transcription activity. SCC9 cells were co-transfected with pGL3-6.1K and wild-type CLOCK- or mutant CLOCK-expressing plasmid. For (**C**–**E**), cells were harvested for analysis of luciferase activity at 24 hr post transfection. Luciferase activity was normalized to protein concentrations, and expressed as arbitrary units. (**F**) ChIP assay in SCC9 cells. The PCR products of the DNA prepared from chromatin immunoprecipitated with IgG and/or antibodies against BMAL1 or CLOCK. (**G**) The resultant DNA of chromatin immunocomplex was subjected to quantitative real-time PCR. For bar graphs, data are means ± SE. n = 5–6. ^††^*P* < 0.01 and ^†††^*P* < 0.001 pGL3-6.1K vs. pGL3-3.2K or pGL3 for the same Zeitgeber time (in **C**); *P < 0.05; **P < 0.01; and ***P < 0.001 ZT19 vs. ZT7 for the same condition (pGL3-6.1K in (**C**)), CLOCK vs. GFP (in **D**) under the same condition (pGL3-6.1K in (**E**)), or BMAL1 or CLOCK vs. IgG (in **G**); ^‡‡^*P* < 0.01 mCLOCK vs. CLOCK under the same condition (in (**E**)).

To explore how clocks control PFKFB3 rhythmicity, luciferase reporter assays were performed in the synchronized SCC9 cells at ZT7 and ZT19, two time points chosen based on the patterns of PFKFB3 and CLOCK oscillation. Luciferase expression was driven by a 6.1 kb fragment of PFKFB3 promoter (pGL3-6.1 K), a 3.2 kb irrelevant sequence (pGL3-3.2 K), or empty promoter sequence (pGL3). The 6.1 kb fragment contained 33 E-boxes and one strict binding site (CACGTGA) (Fig. 4A). The latter is 431 bp upstream of the starter codon ATG of PFKFB3 DNA. In contrast, the 3.2 kb fragment only contained two putative E-boxes and did not have the strict binding site. For the first set of experiments, pGL3-6.1 K, pGL3-3.2 K, and/or pGL3 (Fig. 4B) were transfected into the synchronized SCC9 cells. Compared with that measured in pGL3-transfected control SCC9 cells, luciferase activity in pGL3-6.1 K-transfected cells was markedly increased at both ZT7 and ZT19 (Fig. 4C). However, luciferase activity measured in pGL3-3.2k-transfected cells did not differ significantly from that in control cells at either ZT7 or ZT19. When compared between the two time points, luciferase activity in pGL3-6.1k-transfected cells at ZT7 was significantly higher than that at ZT19. In combination, these results confirm that endogenous circadian clocks drive PFKFB3 rhythmicity in SCC9 cancerous cells by regulating PFKFB3 promoter transcription activity. To address a direct effect of CLOCK on PFKFB3 promoter activity, SCC9 cells were co-transfected with the pGL3-6.1k plasmid and a plasmid expressing BMAL1, CLOCK, or green fluorescent protein (GFP). Also, a group of SCC9 cells were co-transfected with the pGL3-6.1k construct, a BMAL1-expressing plasmid, and a CLOCK-expressing plasmid. As indicated by the luciferase activity, PFKFB3 promoter transcription activity was significantly increased upon overexpression of CLOCK, but not BMAL1 (Fig. 4D). In addition, PFKFB3 promoter transcription activity was not increased upon co-overexpression of BMAL1 and CLOCK. Next, the present study compared the effects of wild-type (WT) CLOCK and CLOCK-Δ19 mutant (mCLOCK). As expected, WT CLOCK increased PFKFB3 promoter transcription activity in pGL3-6.1k-transfected cells but not in pGL3-3.2k-transfected cells (Fig. 4E). Unlike WT CLOCK, mCLOCK did not increase PFKFB3 promoter transcription activity in pGL3-6.1k-transfected cells compared with GFP. Together, these results indicate that CLOCK drives PFKFB3 rhythmicity by stimulating PFKFB3 promoter transcription activity.

To validate whether PFKFB3 promoter region contains genuine E-box(s) to allow its binding to BMAL1, CLOCK, or both, the present study performed the chromatin-immunoprecipitation (ChIP) assay in SCC9 cells. Compared with the negative control, both BMAL1 and CLOCK were capable of binding an area of PFKFB3 promoter (Fig. 4F,G). In addition, the DNA binding sequence for both BMAL1 and CLOCK was identified as the forth upstream putative E-box (Fig. 4A, the underlined symbol of the bottom panel). These results suggest that PFKFB3 promoter region contains at least a genuine E-box.

### Timed PFKFB3 inhibition brings about different consequences on SCC9 cell production of lactate, proliferation, and apoptosis

**Figure 5:**
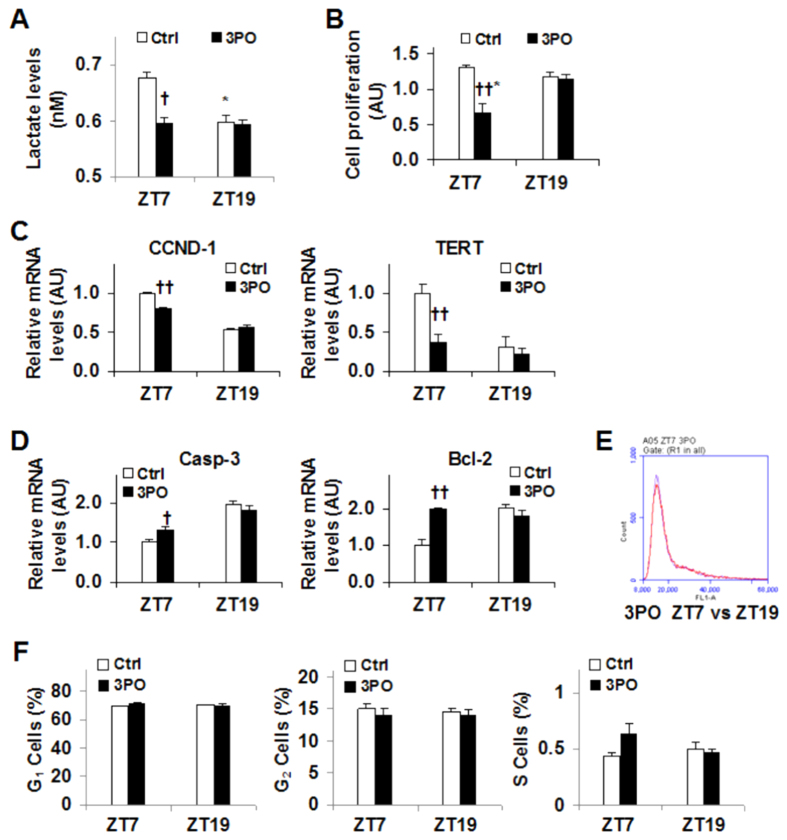
Timed PFKFB3 inhibition causes different consequences on SCC9 cell production of lactate, proliferation, and apoptosis. SCC9 cells were synchronized and treated with 3PO (0.5 μg/ml, in DMSO) or DMSO as control (Ctrl) at 7 hr and/or 19 hr post synchronization (zeitgeber time, ZT and ZT19, respectively). At 48 hr post 3PO/DMSO treatment, conditioned media were collected to measure the concentrations of lactate, and the cells were harvested to analyze proliferation, the expression of genes for proliferation and apoptosis, and cell-cycles. (**A**) Lactate production. (**B**) Proliferation was measured using a CCK8 kit. (**C**) The mRNA levels of the expression of genes for proliferation. (**D**) The mRNA levels of the expression of genes for apoptosis. (**E**) A representative FACS plot of cell-cycle phases of cells treated with 3PO at ZT7 and/or ZT19. (**F**) Quantification of cells in G_1_, G_2_, and S phases in response to timed 3PO treatment. For (**C,D**), the mRNA levels were quantified using real-time PCR and plotted as relative expression. For all bar graphs, data are means ± SE. n = 5–6. ^†^*P* < 0.05 and ^††^*P* < 0.01 3PO vs. Ctrl for the same zeitgeber time; *P < 0.05 ZT7 vs. ZT19 with the same treatment (Ctrl or 3PO).

PFKFB3 is a master regulator of glycolysis, whose increase contributes to cancer cell growth and survivals. The present study measured SCC9 cell production of lactate to assess the effect of timed PFKFB3 inhibition on glycolysis. PFKFB3 inhibition by 3PO did not alter the amount of iPFK2 (data not shown). However, inhibition of PFKFB3 by 3PO at ZT7, a time when both PFKFB3 expression and lactate production were at high levels, caused a significant decrease in lactate production (Fig. 5A). In contrast, PFKFB3 inhibition by 3PO at ZT19, a time when both PFFKB3 expression and lactate production were at relatively low levels, did not further decrease lactate production. These results suggest that 3PO appears to decrease the rates of glycolysis only when glycolysis is at high levels.

The dysregulation of rhythmic PFKFB3 expression exists in cancerous cells. Next, the present study determined the effect of timed PFKFB3 inhibition on parameters related to cell growth. As indicated by the results from the CCK8 assay, PFKFB3 inhibition by 3PO caused a significant decrease in cell proliferation compared with control treatment at ZT7 (Fig. 5B). However, 3PO was not effective at ZT19. In consistency, the mRNA levels of CCND-1 and TERT, two genes that promote cell proliferation, were significantly decreased upon 3PO treatment at ZT7 compared with their respective levels in control cells (Fig. 5C). This decrease, however, was not observed in 3PO-treated cells at ZT19. In contrast, 3PO treatment at ZT7, but not ZT19, caused a significant increase in the mRNA levels of Casp3 and Bcl2, two mediators that stimulate cell apoptosis (Fig. 5D). The present study also examined the effect of timed PFKFB3 inhibition on cell cycles, and did not observe any significant difference (Fig. 5E,F). In combination, these results suggest that PFKFB3 inhibition at its peak expression time generates better efficacy at inhibiting proliferation and stimulating apoptosis of cancer cells.

### Timed PFKFB3 inhibition differentially alters the growth of implanted neoplasms in mice

**Figure 6:**
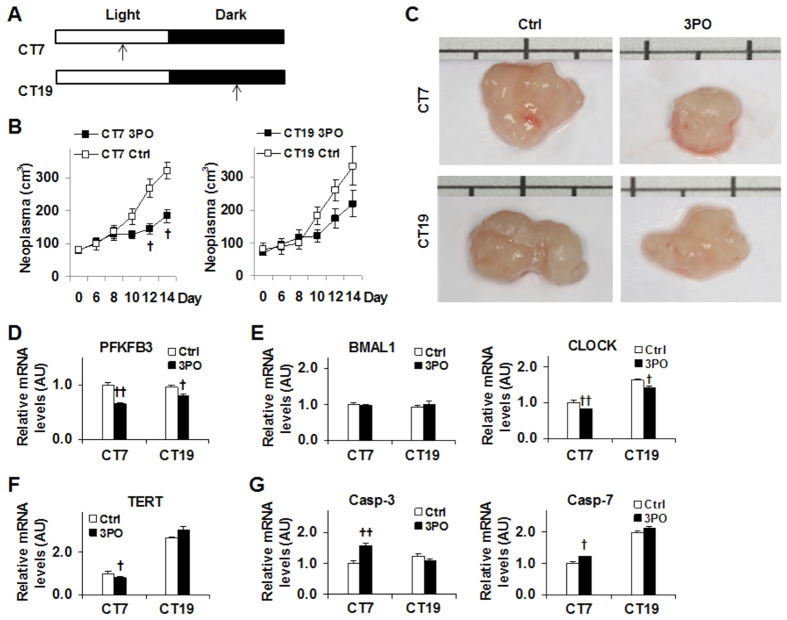
Timed PFKFB3 inhibition differentially influences the growth of implanted neoplasms in mice. Mice were implanted with SCC9 cells on day 0. Immediately after implantation of SCC9 cells, treatment with 3PO (70 mg/kg, in DMSO) or DMSO began at circadian time 7 (CT7, 3 pm) or CT19 (3 am) via an intraperitoneal injection, and continued daily for 14 days. At 6 days post the initial treatment, the volume of the implanted neoplasms was measured every two day for 8 additional days. n = 5. (**A**) Scheme for 3PO treatment. (**B**) The size of implanted neoplasms. (**C**) General appearance of implanted neoplasms. (**D**) The mRNA levels of PFKFB3 expression. (**E**) The mRNA levels of BMAL1 and CLOCK. (**F**) The mRNA levels of TERT. (**G**) The mRNA levels of the expression of genes for apoptosis. For (**D**–**G**), the mRNA levels were quantified using real-time PCR and plotted as relative expression. For bar graphs, data are means ± SE. ^†^*P* < 0.05 and ^††^*P* < 0.01 3PO vs. Ctrl for the same day (in **B**) at the same time (CT7 or CT19 in (**D**–**G)**).

To address if timed PFKFB3 inhibition generates different effects on cancer growth, SCC9 cell-implanted mice were treated with 3PO separately at circadian time 7 (CT7) and CT19 (Fig. 6A). For mice assigned with light-time (CT7) treatment, 3PO significantly reduced the size of implanted neoplasms after treatment for 12 days in comparison to control (DMSO) treatment (Fig. 6B,C). However, 3PO treatment at dark-time (CT19) only caused an insignificant decrease in the size of implanted neoplasms. These results were in agreement with the results obtained from cultured SCC9 cells upon timed PFKFB3 inhibition (Fig. 5). In the isolated neoplasms, the mRNA levels of PFKFB3 were decreased to a greater extent at CT7 than at CT19 (Fig. 6D). In addition, the mRNA levels of CLOCK also were decreased to a greater extent at CT7 than at CT19 whereas the mRNA levels of BAMAL1 did not differ among all neoplasms (Fig. 6E). With regard to the mediators of cell proliferation and apoptosis, 3PO treatment significantly decreased TERT mRNAs at CT7, but not at CT19 compared with control treatment (Fig. 6F). In contrast, 3PO treatment significantly increased the mRNA levels of Casp-3 and Casp-7 at CT7, but not CT19, compared with control treatment (Fig. 6G). Taken together, these results suggest that timed PFKFB3 inhibition generates different outcomes on cancer growth, which is attributable to differential effects of timed PFKFB3 inhibition on cancer cell proliferation and apoptosis.

## Discussion

PFKFB3 expression was increased at both the mRNA and protein levels in human tongue cancers. This finding was consistent with a role for PFKFB3 in supporting cancer growth. Of interest, the expression of key clock genes was either down-regulated, e.g., BMAL1, CLOCK, PER1, PER2, and PER3, or up-regulated, e.g. CYR1 and CRY2, in tongue cancer samples in relative to that in normal control samples. In combination, these results indicate that PFKFB3 up-regulation is associated with circadian clock dysregulation. However, the down-regulation of BMAL1 and CLOCK expression and the up-regulation of PFKFB3 expression in tongue cancer samples do not necessarily imply that BMAL1 and/or CLOCK down-regulation leads to increased PFKFB3 expression. This is likely because clinical samples were collected at different times of the dates when oral surgeries were performed. Considering this, cultured tongue cancer cells and control cells were synchronized and used to confirm the relationship between BMAL1/CLOCK and PFKFB3. The rhythmic expression of PFKFB3, as well as CLOCK in cancerous cells was distinct from that in control cells, demonstrating the existence of circadian clock dysregulation that was closely associated with cancer development and progression (see below). Of importance, PFKFB3 expression was peaked at a time 4 hr behind of peaked CLOCK expression and was not correlated with BMAL1 expression. Thus, CLOCK appears to mainly control the rhythmic expression of PFKFB3.

To address a mechanistic link between PFKFB3 and CLOCK, the present study analyzed the sequence of a 6.1 kb fragment of PFKFB3 promoter and indicated 33 putative E-boxes including one strict CLOCK/BMAL1 binding site. This led to the postulation that CLOCK binds PFKFB3 promoter and initiates/enhances its transcription activity, thereby bringing about rhythmic PFKFB3 expression. As supporting evidence, the transcription activity of PFKFB3 promoter at ZT7 was markedly higher than that at ZT19 in cancerous cells, a rhythmic pattern that was nearly identical to that of PFKFB3 expression. In addition, overexpression of WT CLCOK, but not mutant CLOCK, increased the transcription activity of PFKFB3 promoter. These results not only indicate that CLOCK stimulates PFKFB3 expression by binding PFKFB3 promoter and increasing its transcription activity, but more importantly demonstrate that CLOCK stimulation of rhythmic PFKFB3 expression is enhanced in cancer cells. As confirmatory evidence, the present study validated one genuine E-box that binds BMAL1, CLOCK, or both. To be noted, while CLOCK overexpression enhanced PFKFB3 promoter transcription activity in SCC9 cells, BMAL1 overexpression did not. These results, however, do not necessarily indicate that BMAL1 negatively influences the effect of CLOCK on binding and enhancing PFKFB3 promoter transcription activity; given that BMAL1 and CLOCK commonly form a heterodimer to initiate the transcription of clock genes including PER1, PER2, PER3, CYR1 and CRY2. One likely explanation for the results of the reporter assays in this study is that BMAL1, when overexpressed alone, was not sufficient enough to enhance PFKFB3 promoter transcription activity. Additionally, when co-overexpressed, both BMAL1 and CLOCK likely were expressed at the levels below their respective thresholds for enhancing PFKFB3 promoter transcription activity. Alternatively, BMAL1 may recruit certain binding protein(s) that inhibit CLOCK actions on enhancing PFKFB3 promoter transcription activity. Regardless of the exact interplay between BMAL1 and CLOCK in SCC9 cells, it is certain that at least one genuine E-Box site enables PFKFB3 rhythmic expression.

Increased PFKFB3 expression is seen in a number of human cancers^31^,^34^. Because of this, PFKFB3 inhibition is considered as a potential anti-cancer therapy^33^,^35^. Indeed, an ongoing clinical trial is examining the efficacy of a PFKFB3 inhibitor at treating lung cancer^36^. Given this, how to effectively inhibit PFKFB3 could be key to a better anti-cancer treatment. In the present study, circadian clock control of PFKFB3 expression was established as discussed above, and served as the basis for the present study to examine the effects of timed PFKFB3 inhibition. Of significance, PFKFB3 inhibition at ZT7, but not at ZT19, decreased cancer cell production of lactate. These results indicate the importance of timing to PFKFB3 inhibition and glycolysis in the context of cancer growth and survivals. Consistently, PFKFB3 inhibition at ZT7, but not at ZT19, reduced cancer cell proliferation. In addition, PFKFB3 inhibition at ZT7, but not ZT19, decreased the expression of anti-apoptotic genes and increased the expression of pro-apoptotic genes. Considering that PFKFB3 expression was peaked at ZT7 in synchronized SCC9 cells, it is very likely that inhibiting PFKFB3 at its peaked expression is more effective. This, in turn, led to improved efficacy of PFKFB3 inhibition at reducing the proliferation of cancer cells, which appeared to be due to improved efficacy at reducing the expression of anti-apoptotic genes and at increasing the expression of apoptotic enzymes. Clearly, circadian clocks critically control PFKFB3 expression, thereby cancer cell proliferation. In other words, PFKFB3 appears to serve as a mediator that links circadian clocks and the growth of cancer cells.

The significance of timed PFKFB3 inhibition is further manifested by the results from the mice study. Notably, treatment of mice with 3PO at a fixed time point of daytime (CT7) significantly reduced the size the implanted neoplasms whereas the same treatment at a fixed time point of nighttime (CT19) only caused an insignificant decrease in the size of implanted neoplasms. As suggested by the results from cancer cells upon timed PFKFB3 inhibition, it is very likely that 3PO directly inhibited the PFKFB3 of implanted cancer cells at CT7, thereby increasing apoptosis of the implanted cancer cells. In support of this, the expression of Casp-3 and Casp-7 of implanted neoplasms upon 3PO treatment at CT7 was significantly higher than that in implanted neoplasms upon 3PO treatment at CT19. Additionally, 3PO treatment at CT7 was more effective than at CT19 in terms of decreasing the expression of TERT, which also appeared to contribute to the observed decrease in the size of implanted neoplasms. It should also be pointed out that 3PO treatment at CT7, but not at CT19, was more effective at decreasing the expression of PFKFB3 and CLOCK in implanted neoplasms. Considering the stimulatory effect of CLOCK on PFKFB3 expression, it is likely that 3PO treatment also acted through reducing CLOCK expression to decrease PFKFB3 expression, thereby amplifying its anti-cancer effect. However, future study is needed to elucidate the mechanisms underlying the effect of PFKFB3 inhibition on reducing CLOCK expression, and to determine the extent to which other CLOCK-associated mechanisms contribute to the anti-cancer effect of 3PO in a timed manner. Lastly, 3PO treatment at different time points may generate differential effects on host cells, which in turn differentially influenced the growth of implanted neoplasms. In this regard, the contribution from host cells to the overall outcomes of PFKFB3 inhibition on the implanted neoplasms cannot be ruled out. Currently, nearly all anti-cancer chemotherapies produce significant unwanted side-effects, e.g., killing normal cells. Considering that cancerous cells and normal cells commonly display differences in rhythmicity of CCG, timed anti-cancer therapies that are based on rhythmicity CCG of cancer cells would reduce un-wanted sides effects that are caused by killing normal cells. In fact, timed PFFKB3 inhibition could be a perfect example of such anti-cancer therapy.

In summary, the present study provides compelling evidence, for the first time, to support a link between circadian clock dysregulation and increased PFKFB3 expression in the context of cancer bio-physiology. Of significance, CLOCK stimulated PFKFB3 expression through increasing the transcription activity of PFKFB3 promoter, which appeared to be responsible for distinct PFKFB3 rhythmic expression in cancer cells. Based on this link, the effects of timed PFKFB3 inhibition were examined in cultured cancer cells and in mice implanted with cancer cells. The pertinent results support a role for PFKFB3 in the control of cancer growth by responding to circadian clock outputs. Thus, timed PFKFB3 inhibition could improve the efficacy of anti-cancer therapy.

## Methods

### Collection and preparation of clinical samples

Subjects with or without tongue cancers were from both outpatient and in-patient services of the Department of Stomatology of Union Hospital of Tongji Medical College of Huazhong University of Science and Technology (Wuhan, China) during the time period between year 2013 and year 2014. During their visits, general parameters obtained from physical examination were recorded and documented by physicians and dentists who saw the patients. In addition, blood samples, collected during patients’ visits, were used for general biochemical lab assays, which were also documented by the Department of Stomatology. During oral surgeries, tongue tissues were removed from each subject and split into two parts. One part was immediately fixed in 4% paraformaldehyde for histological validation and immunohistochemical staining, and the other part was frozen stored at −80 °C before total RNA isolation and RT-PCR assay using methods detailed below. Based on histological changes, the present study collected samples from human tongue squamous cell carcinoma (hTSCCs) (n = 27), matched tumor adjacent (surrounding tissues) (n = 27), and benign tissues (n = 28). Detailed clinical characteristics were provided in Table 1. In addition, informed consent was obtained from all subjects, and the methods involving human subjects were performed in accordance with the standard practice procedures, regulations, and guidance and were approved by the Union Hospital of Tongji Medical College.

### Cell Culture

Human tongue carcinoma SCC9 cells and control KC cells were commercially obtained from ATCC. SCC9 and KC cells were maintained in Dulbecco’s Modified Eagle’s/Ham’s F12 Medium (Sigma, MO, USA) supplemented with 10% heated-inactivated fetal bovine serum, 0.5% penicillin, 0.5% streptomycin, 1.2 g/L sodium bicarbonate, 400 μg/L hydrocortisone in a humidified 5% CO2 atmosphere at 37 °C. Culture medium was changed every 3 days.

### Oscillation of PFKFB3 and core clock gene expression in SCC9 cells

SCC9 and KC cells were seeded (1 × 10^5^/well) into 6-well plates with cell culture medium as described above. After incubation to reach 70% to 100% confluence, SCC9 and KC cells were subjected to synchronization^37^. Briefly, SCC9 and KC cells were incubated in DMEM/F12 containing 50% horse serum for 2 hr, followed by incubation in DMEM/F12 with 5% FBS for an additional hr to complete synchronization, when the time was set as zeitgeber time 0 (ZT0). Some synchronized cells were harvested every 6 hr after ZT0 during a 30-hr time period and some cells were harvested at 1, 5, 9, 13, 17, and 21 hr after synchronization (ZT1, ZT5, ZT9, ZT13, ZT17, and ZT21). After harvest, total RNA was prepared and used for further analyses.

### Transfection and luciferase assays

For reporter assays, three constructs were used, in which luciferase expression was driven by a 6.1 kb fragment of PFKFB3 promoter (pGL3-6.1 K), a 3.2 kb irrelevant sequence (pGL3-3.2 K), or empty promoter sequence (pGL3). Prior to transection of SCC9 cells, three types of plasmids were prepared as previously described^38^,^39^. For co-transfection experiments, a plasmid expressing BMAL1, CLOCK, or green fluorescent protein (GFP) was prepared similarly. In brief, SCC9 cells were seeded into 6-well plates and synchronized as described above. After synchronization, some cells were transfected with pGL3-6.1 K, pGL3-3.2 K, or pGL3 using Lipofectamine Plus (Invitrogen) in DMEM/F12 medium at ZT7 and ZT19 respectively, according to the manufacturer’s instructions. After transection and incubation with normal growth media for an additional 24 hr, the transfected cells were harvested for determination of luciferase activity using a Dual Luciferase Reporter Assay System (Promega, Madison, WI). To address CLOCK control of PFKFB3 promoter transcription activity, some SCC9 cells, without synchronization, were co-transfected with pGL3-6.1k and a plasmid expressing BMAL1, CLOCK, CLOCKΔ19 mutant (mCLOCK), or GFP. In an additional group of SCC9, pGL3-6.1k was co-transfected with a BMAL1-expressing plasmid and a CLOCK-expressing plasmid. After transfection and incubation for an additional 24 hr, the cells were harvested and subjected to luciferase assays.

### Chromatin immunoprecipitation assays

SCC9 cells were cultured as described above and subjected to chromatin immunoprecipitation (ChIP) assays following a standard procedure provided by a kit (Catalog # 17–371, Millipore). Briefly, cells were crosslinked with medium containing 1% formaldehyde for 15 min at room temperature. After crosslinking, cells were washed and resuspended in a lysis buffer containing protease inhibitors. The samples were sonicated to generate fragments of average length of 200–500 bp. Cellular debris were removed by centrifugation at 4 °C for 10 min (10, 000 g) and supernatant was diluted 5-fold in dilution buffer supplemented with protease inhibitors. The samples were further prepared and incubated in the presence of different primary antibodies for overnight at 4 °C. Immune complexes were recovered by centrifugation at 4 °C for 1 min (500 g). After several washes, all samples, including inputs, were incubated at 65 °C for 4 hr in the presence of proteinase K. After reversing crosslinks of protein/DNA complexes, DNA was purified and subjected to regular PCR and quantitative real-time PCR. Primer sequences are available upon request.

### Timed PFKFB3 inhibition and measurement of lactate production, proliferation, apoptosis, and cell- cycle in SCC9 cells

SCC9 cells were seeded into 96-well plates (5000 cells/well) or 6-well plates (1 × 10^5^cells/well), and synchronized as described above. After synchronization, SCC9 cells were treated with PFKFB3 inhibitor 3PO (0.5 μg/ml, in DMSO) or DMSO separately at ZT7 and ZT19. To analyze glycolysis, conditioned SCC9 media were collected to measure the concentrations of lactate. To analyze cell proliferation, after treatment for 48 hr, the medium of SCC9 cells seeded in 96-well plates was replaced with normal culture medium containing 10% CCK8 kit solution and incubated at 37 °C for an additional 1.5 hr. The absorbance of CCK8 solutions was measured with a plate reader at 450 nm wave-length with 5 replicates per group. For cells in 6-well plates, STAT60 regents and/or lysis buffer were added for preparation of RNA and protein samples, respectively, as previously described^38^,^39^. To analyze apoptosis, RT-PCR was performed to quantify the mRNA levels of anti-apoptosis genes, such as CCND-1 and TERT, and pro-apoptosis genes such as Casp-3, Casp-7, and Bcl2. Some cells with identical treatments were subjected to flow cytometry to analyze cell cycles as described below.

### Cell cycle and flow cytometry assay

SCC9 cells were synchronized and treated as described above. After treatment, the cells were digested with 1× trypsin and fixed in the cold 100% ethanol. After incubation for 20 min, the cells were washed with PBS, and stained with staining solution (5% Propidium Iodide, 10% DNase-free RNase A, 10% Sodium citrate, 0.2% Triton X-100, and 74.8% Nanopure water). After incubation for 30 min at room temperature, the cells were added with 20 μl sodium chloride and subjected to FACS analyses using BD Accuri™ C6 flow cytometer (BD Biosciences, San Jose, CA).

### Timed PFKFB3 inhibition in mice

Twenty male mice, at 3–4 weeks of age, were obtained from Fukang Company (Beijing, China), and housed at the Animal Experimental Center of Union Hospital, Tongji Medical College of Huazhong University of Science and Technology (Wuhan, Hubei, China) with 12-h light/dark cycles and fed antibiotic-free food and water ad libitum. After acclimation, mice were injected with SCC9 cells (5 × 10^6^ cells/ml, in 1 ml PBS) into the dorsal. When the size of implanted neoplasms grew to 60–80 cm^3^, the implanted mice were treated with 3PO (70 mg/kg/d, in DMSO) or DMSO at circadian time CT7 (3:00 pm) or CT19 (3:00 am) via intraperitoneal injections. Six days after the initial treatment, the volume of the implanted neoplasms was measured every two day for 8 additional days. On day 14 after the treatment period, all the mice were euthanized for harvest of neoplasm and liver tissues. Each tissue was cut into two pieces. One piece was fixed immediately in 4% paraformaldehyde for histopathology validation, and the other piece was stored in RNAlater RNA Stabilization Reagent (QIAGEN; Valencia, CA) before RNA preparation for quantitative real-time PCR. The animal study protocol was reviewed and approved by the Institutional Animal Care and Use Committee of Tongji Medical College. In addition, all experiments were performed in accordance with relevant guidelines and regulations.

### Histological and immunohistochemical analyses

The paraffin-embedded tissue blocks were cut into sections of 5 μm thickness and stained with H&E. In addition, sections were stained for the expression of iPFK2 with rabbit anti-iPFK2 antibodies (1:100) (Sanying, Wuhan, China).

### RNA isolation, reverse transcription, and real-time PCR

The total RNA was isolated from frozen human tongue tissues, cultured SCC9 cells, and implanted neoplasms from mice. Reverse transcription was performed using the GoScript™ Reverse Transcription System (Promega, Madison, WI) and real-time PCR analysis was performed using SYBR Green (LightCycler^®^ 480 system; Roche Life Science, Indianapolis, IN). The mRNA levels were analyzed for PFKFB3, BMAL1, CLOCK, PER1, PER2, PER3, CRY1, CRY2, CCND-1, TERT, Casp-3, Casp-7, and/or Bcl2. Results were normalized to 18 s ribosomal RNA and plotted as relative expression to the average of normal tissue, SCC9 cells at ZT1, or DMSO (Ctrl)-treated cells at ZT7 or mice CT7, which was set as 1. Primer sequences are available upon request.

### Statistical Methods

Numeric data are presented as means ± SE (standard error) except clinical parameters detailed in Table 1. Two-tailed Student’s t tests and/or two tailed ANOVA tests were used for statistical analyses. Differences were considered significant at the *P* < 0.05.

## Additional Information

**How to cite this article**: Chen, L. *et al.* PFKFB3 Control of Cancer Growth by Responding to Circadian Clock Outputs. *Sci. Rep.* **6**, 24324; doi: 10.1038/srep24324 (2016).

## Figures and Tables

**Table 1:** Clinical characteristics of subjects with or without tongue cancers.

	hTSCCs (n = 27)	Matched tumor adjacent (n = 27)	Benign tissue (n = 28)
Gender [n (%)]			
Male	18(66.7)	18(66.7)	16(57.1)
Female	9(33.3)	9(33.3)	12(42.9)
BMI^1^	26.3(5.4)	26.3(5.4)	27.8(7.2)
Age (years)^1^	52.6(9.15)	52.6(9.15)	48.4(13.8)
Fasting blood glucose (mM)^1^	5.40(1.00)	5.40(1.00)	4.95(0.52)
Staging [n (%)]			
T1/2	12(44.4)	12(44.4)	
T3/4	15(55.6)	15(55.6)	
Lymph node status [n (%)]			
Positive	10(37.0)	10(37.0)	
Negative	17(63.0)	17(63.0)	
Differentiation [n (%)]			
Well	9(33.3)	9(33.3)	
Moderate	13(48.2)	13(48.2)	
Poor	5(18.5)	5(18.5)	

hTSCCs, human tongue squamous cell carcinoma.

^1^Data are means (SD).
